# RNA Binding Properties of SOX Family Members

**DOI:** 10.3390/cells13141202

**Published:** 2024-07-16

**Authors:** Seyed Mohammad Ghafoori, Ashish Sethi, Gayle F. Petersen, Mohammad Hossein Tanipour, Paul R. Gooley, Jade K. Forwood

**Affiliations:** 1 School of Dentistry and Medical Sciences, Charles Sturt University, Wagga Wagga, NSW 2678, Australia; sghafoori@csu.edu.au; 2 Department of Biochemistry and Pharmacology, Bio21 Molecular Science and Biotechnology Institute, University of Melbourne, Parkville, VIC 3010, Australia; sethia@ansto.gov.au (A.S.); mohammadhossein.tanipour@unimelb.edu.au (M.H.T.); prg@unimelb.edu.au (P.R.G.); 3 Australian Nuclear Science Technology Organisation, The Australian Synchrotron, 800 Blackburn Rd., Clayton, VIC 3168, Australia; 4 Gulbali Institute, Charles Sturt University, Wagga Wagga, NSW 2678, Australia; gpetersen@csu.edu.au

**Keywords:** SOX, HMG-box, RNA binding

## Abstract

SOX proteins are a family of transcription factors (TFs) that play critical functions in sex determination, neurogenesis, and chondrocyte differentiation, as well as cardiac, vascular, and lymphatic development. There are 20 SOX family members in humans, each sharing a 79-residue L-shaped high mobility group (HMG)-box domain that is responsible for DNA binding. SOX2 was recently shown to interact with long non-coding RNA and large-intergenic non-coding RNA to regulate embryonic stem cell and neuronal differentiation. The RNA binding region was shown to reside within the HMG-box domain; however, the structural details of this binding remain unclear. Here, we show that all SOX family members, except group H, interact with RNA. Our mutational experiments demonstrate that the disordered C-terminal region of the HMG-box domain plays an important role in RNA binding. Further, by determining a high-resolution structure of the HMG-box domain of the group H family member SOX30, we show that despite differences in RNA binding ability, SOX30 shares a very similar secondary structure with other SOX protein HMG-box domains. Together, our study provides insight into the interaction of SOX TFs with RNA.

## 1. Introduction

**Figure 1:**
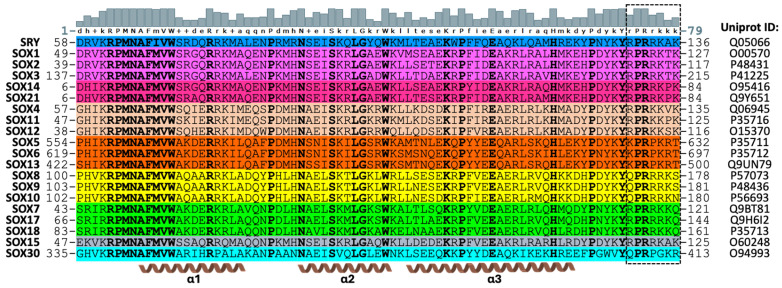
SOX family members share a similar HMG-box domain. Amino acid sequence alignment of the HMG-box domains from human SOX family members, coloured by group and numbered as per full-length UniProt sequences; conserved residues are shown in bold. A conservation bar is shown at the top of the alignment in grey, numbered as per HMG-box numbering; upper case = conserved residue, lower case = most common residue. The three α-helices of the HMG-box domain are indicated at the bottom of the alignment. The black dashed box highlights the region proposed to be critical for RNA binding. The alignment was produced using UGENE software version 36.0 [15].

Sex-determining region Y (SRY) was the founding member of a 20-member family of transcription factors (TFs) known as SRY-related high mobility group (HMG)-box (SOX) proteins. SOX proteins play crucial roles in various biological processes, including development, organogenesis, cell fate, and homeostasis [1,2,3,4,5]. All SOX family members share a common 79-residue HMG-box domain, with >50% sequence similarity to the SRY HMG-box (Figure 1) [6]. The L-shaped HMG-box comprises three α-helices: α1 and α2 that form one arm of the L (major wing), and α3 that forms the other arm of the L (minor wing) [7]. The HMG-box is responsible for binding and bending DNA [8], specifically at the consensus site (A/T)(A/T)CAA(A/T)G [9]. Unlike most other TFs, the HMG-box of SOX proteins binds to the minor groove of DNA, inducing a bend of 60–70° due to a wedge formed by the conserved Phe-Met (FM) dipeptide positioned on α1 that intercalates between bases and kinks the DNA [10]. The SOX HMG-box also features three key regions for nuclear localisation: two basic regions for nuclear import at the distal ends of the HMG-box domain and one leucine-rich nuclear export signal [11,12,13,14]. These regions regulate nucleocytoplasmic trafficking of SOX proteins, resulting in varying subcellular distribution throughout development.

Based on phylogenetic analysis of the HMG-box domain, SOX proteins are divided into nine groups (A, B1, B2, C, D, E, F, G, and H). SRY is the only member of the SOXA group, with an essential role in sex determination [6]. SOXB1 group members (SOX1, SOX2, and SOX3) play important roles in neural development, specifically formation of the neural primordium [16], proliferation and differentiation of neural stem cells during embryogenesis [17], and regulation of the maintenance/proliferation of adult neural stem cells during neurogenesis [18], as well as lens development, eye morphogenesis [19,20,21], inner ear development, and sensory hair cell differentiation [22]. SOXB2 group members (SOX14 and SOX21) also play a role in neural differentiation, negatively repressing the downstream Notch signalling molecule *HES5* to promote neurogenesis and differentiation of neural stem cells [23]. Members of the SOXC group (SOX4, SOX11, and SOX12) contribute to nervous system development [24] and retinal differentiation [25]. In addition to their role in neural development [26], SOXD group members (SOX5, SOX6, and SOX13) play a critical role in chondrocyte differentiation and cartilage formation [27,28]. The main function of SOXE group members (SOX8, SOX9, and SOX10) is sex determination [29], while SOXF group members (SOX7, SOX17, and SOX18) play a crucial role in cardiac, vascular, and lymphatic development [30]. Finally, the functions of SOXG and SOXH group members (SOX15 and SOX30, respectively) are not yet fully elucidated; however, they have been shown to play roles in cancer prevention and apoptosis [31,32].

It has been demonstrated that some TFs possess the ability to bind both DNA and RNA [33,34,35]. For instance, overexpression of p53 has been shown to suppress *mdmx* mRNA translation by binding to the 5′ untranslated region of the *mdmx* mRNA [36]. Similarly, Ubx can bind RNA, regulating mRNA expression and co-transcriptional splicing [37], and YY1 can bind gene regulatory elements and their associated RNA, contributing to the maintenance of some TFs at gene regulatory elements [34]. Recently, it was identified that SOX2 can also bind both DNA and RNA. RNA immunoprecipitation experiments demonstrated an association between SOX2 and long non-coding RNA (lncRNA)_ES1 (AK056826) and lncRNA_ES2 (EF565083) for regulation of embryonic stem cell (ESC) pluripotency [38]. A further study found that SOX2 binds to lncRNA_ES2 through its DNA-binding HMG-box, with both high affinity and low sequence specificity [39]. Studies have also shown that the lncRNA *RMST* associates with SOX2 and regulates neuronal differentiation [40], and the large-intergenic non-coding RNA 1614 interacts with SOX2 to mediate transcriptional silencing and maintain ESC pluripotency [41]. One study has demonstrated that Sox2 binds RNA via a 60 amino acid region directly after the HMG-box, with a preference for GC-rich RNA sequences [42], whereas another study has linked the RNA binding ability of TFs, including SOX2, to an Arginine Rich Motif (ARM)-like domain encompassing the C-terminal end of the HMG-box and residues directly after this [43]. Due to a lack of structural and molecular information detailing how SOX proteins bind RNA, here we examine the RNA binding properties across a range of SOX family members and demonstrate that binding resides in the HMG-box C-terminal region.

## 2. Materials and Methods

### 2.1. Protein Expression and Purification

The HMG-box domains from one member of each SOX group were cloned into different vectors (Table S1) and transformed into BL21 (DE3) pLysS *E. coli* cells (ThermoFisher Scientific, Waltham, MA, USA) for protein expression. Cells were grown in 5 mL of Luria–Bertani (LB) media (tryptone 10 g/L, yeast extract 5 g/L, sodium chloride 10 g/L) supplemented with the appropriate antibiotic at 37 °C until the OD600 reached 0.6–0.8. For large-scale protein expression, 1 mL of starter culture was added to 1 L of expression media (tryptone 10 g/L, yeast extract 5 g/L, dipotassium hydrogen phosphate 8.7 g/L, potassium dihydrogen phosphate 6.8 g/L, sodium sulphate 0.71 g/L, magnesium sulphate 0.24 g/L, glycerol 5 g/L, glucose 0.5 g/L, lactose 2 g/L) with the appropriate antibiotic, and expression was induced for 36 h at room temperature using the auto-induction method described previously [44]. For the SOX6 HMG-box domain, expression was induced for 24 h at 18 °C using the IPTG induction method described previously [45], using IPTG at 1 mM. Cells were harvested at 6400 RCF for 20 min and resuspended in low imidazole phosphate buffer (50 mM phosphate buffer pH 8.0, 300 mM sodium chloride, 20 mM imidazole). Prior to purification, cells were lysed using three freeze–thaw cycles [46] and treatment with 0.5 mg DNaseI (Sigma-Aldrich, St. Louis, MO, USA) and 20 mg lysozyme (Sigma-Aldrich, St. Louis, MO, USA) for 45 min at room temperature. Lysate was injected onto a HisTrap 5 mL column (Cytiva, Marlborough, MA, USA) using low imidazole phosphate buffer, followed by washing with 15 column volumes (CVs) of the same buffer. The sample was eluted with high imidazole phosphate buffer (50 mM phosphate buffer pH 8.0, 300 mM sodium chloride, 500 mM imidazole) using a gradient elution for 5 CVs, followed by 5 CVs of 100% high imidazole phosphate buffer. Eluted fractions were pooled and split into three tubes, of which one was treated with 0.5 mg DNaseI, one was treated with 0.5 mg RNaseA (Sigma-Aldrich, St. Louis, MO, USA), and one was left untreated, prior to incubation at 4 °C on a roller overnight. Analytical gel filtration was performed with 1 mL of each sample on a Superdex 200 pg 10/300 GL column (Cytiva, Marlborough, MA, USA) using gel filtration buffer (50 mM tris, 125 mM sodium chloride, pH 8.0).

### 2.2. Protein/RNA Characterisation

Select analytical gel filtration peak fractions were run on precast 4–12% polyacrylamide Bis-Tris gels (ThermoFisher Scientific, Waltham, MA, USA) using Bolt MES SDS Running Buffer (ThermoFisher Scientific, Waltham, MA, USA) (to visualise protein) and 1.5% agarose gels containing GelRed (Sigma-Aldrich, St. Louis, MO, USA) (1 μL/100 mL) using tris-boric acid (TB) buffer (45 mM tris, 45 mM boric acid), pH 8.5 (to visualise RNA). Polyacrylamide gels were stained with Coomassie blue (0.2% Coomassie brilliant blue, 10% ethanol, 10% glacial acetic acid) and destained (10% ethanol, 10% glacial acetic acid) overnight. Gels were imaged using a Bio-Rad Gel Doc XR+ Imaging System (Bio-Rad Laboratories, Hercules, CA, USA) and images were processed using Image Lab Software (version 6.0.1, Bio-Rad Laboratories, Hercules, CA, USA) and Adobe Photoshop (version 24.0, Adobe, San Jose, CA, USA).

### 2.3. Electrophoretic Mobility Shift Assay (EMSA)

Select ssDNA (Integrated DNA Technologies, Coralville, IA, USA; Table S2) (10 μL of 100 μM) were mixed with SOX proteins (10 μL of 100 μM) and incubated at room temperature for 15 min. Samples were supplemented with 5 μL of 50% glycerol and run on a 1.5% agarose gel containing GelRed (1 μL/100 mL) for 75 min at 70 V in TB buffer, pH 7.4. The gel was imaged, stained with Coomassie blue, destained, and imaged again. Gels were imaged using a Bio-Rad Gel Doc XR+ Imaging System and images were processed and colour-edited using Adobe Photoshop [46].

### 2.4. Fluorescence Polarisation

Two-fold serial dilutions of 20 µM SOX proteins (RNAse-treated) were titrated across 23 wells of a black Fluotrac microplate (Greiner Bio-One, Kremsmünster, Austria) and incubated with 80 nM 3′ FAM-labelled RNA (Integrated DNA Technologies, Coralville, IA, USA; Table S2). Wells were made up to a total volume of 200 µL with gel filtration buffer and fluorescence polarisation was measured using a CLARIOstar Plus plate reader (BMG Labtech, Ortenberg, Germany). Assays were performed in triplicate and included a no protein control used for gain adjustment. Data were analysed in GraphPad Prism (version 10.2.2, GraphPad, San Diego, CA, USA) using non-linear regression assuming one site-specific binding.

### 2.5. Nuclear Magnetic Resonance (NMR)

#### 2.5.1. Expression and Purification

The SOX17 HMG-box domain was expressed in BL21 (DE3) pLysS *E. coli* cells using the autoinduction method [44]. For labelling with ^13^C and ^15^N isotopes, cells were grown in N-5052 [47] supplemented with 3 g/L of D-[^13^C] glucose (Sigma-Aldrich, St. Louis, MO, USA) and 1 g/L of ^15^NH_4_Cl (Sigma-Aldrich, St. Louis, MO, USA) as the sole sources of carbon and nitrogen, respectively. Cells were grown at 37 °C to an OD600 of 0.6–0.7, transferred to 16 °C, and induced (0.4 mM IPTG with shaking overnight at 225–230 rpm). Protein was purified as above, and stored at −80 °C for future use.

#### 2.5.2. NMR Spectroscopy

NMR experiments were performed at 25 °C on a 700 MHz Bruker Avance HDIII spectrometer (Bruker, Billerica, MA, USA) equipped with a triple resonance cryoprobe, using protein dissolved in size exclusion chromatography (SEC) buffer (50 mM tris, 300 mM sodium chloride, 7 mM DTT, pH 8.0). Backbone resonances (^13^Cα, ^13^Cβ, ^13^C’, ^15^N, and NH) of residues were assigned from 3D HNCACB, HN(CO)CACB, HNCO, and HN(CA)CO experiments acquired using non-uniform sampling (NUS). For NUS, sampling schedules were generated using Poisson-gap sampling with 10% of the total number of points collected for all 3D NMR experiments [48]. Spectra were reconstructed with compressed sensing algorithms using qMDD [49] and processed using NMRPipe [50], and data were analysed in NMRFAM-SPARKY [51]. To monitor binding of a 12-mer ssDNA (Integrated DNA Technologies, Coralville, IA, USA; Table S2) to SOX17, 2D ^15^N,^1^H Heteronuclear Single Quantum Coherence (HSQC)-monitored titrations (2048 × 256 data points) were conducted using 100 µM of ^15^N-labelled SOX17 with an increasing concentration (25–100 µM) of 12-mer ssDNA. During titrations, the NMR sample volume was kept within a variation of 10%. The average chemical change was determined from Δδ ppm = √[(Δδ^1^HN)^2^ + (0.15 × Δδ^15^N)^2^] [52].

### 2.6. Crystallisation and Structure Determination

The SOX30 HMG-box domain was cloned, expressed, and purified using nickel affinity chromatography, as described above. Fractions were pooled and further purified by SEC on a Superdex 200 pg 26/600 column (Cytiva, Marlborough, MA, USA) using SEC buffer. Protein was concentrated using an Amicon 10 kDa molecular weight cutoff centrifugal filter (Merck Millipore, Burlington, MA, USA) to 31 mg/mL, aliquoted, and stored at −80 °C. Crystals were produced using the hanging drop vapour diffusion method over 300 μL of reservoir solution. Needle-shaped crystals formed in 0.1 M sodium acetate, 2 M ammonium sulphate, pH 4.6, in 5–7 days. X-ray diffraction data were collected at the Australian Synchrotron on the MX2 beamline using an Eiger 16M detector. iMosflm was used for data reduction and integration [53]. Aimless was used for merging, space group assignment, and scaling, with selection of 5% reflections for Rfree calculations [54]. PhaserMR was used for molecular replacement using PDB ID: 1O4X as the search model [55], and Phenix was used for refinement [56]. Coot was used for modelling [57]. The final model has been validated and deposited in the Protein Data Bank with PDB ID: 7JJK.

## 3. Results and Discussion

### 3.1. RNA Binding Properties of SOX Proteins Extend to All Family Members Except Group H

Based on reports that SOX2 binds both DNA and RNA through its HMG-box domain [39], we investigated whether this RNA binding property extends to other SOX family members. The HMG-box domains of representative SOX proteins from each of the nine groups (Figure 1) were cloned, expressed, purified, and tested for their ability to bind RNA. Our initial assay relied on the ability of SOX HMG-box proteins to co-purify with nucleic acid. Following affinity purification, SOX proteins were either left untreated or treated with DNase or RNase, before further purification on an analytical gel filtration column. Fractions were analysed by both SDS-PAGE and agarose gel electrophoresis.

**Figure 2:**
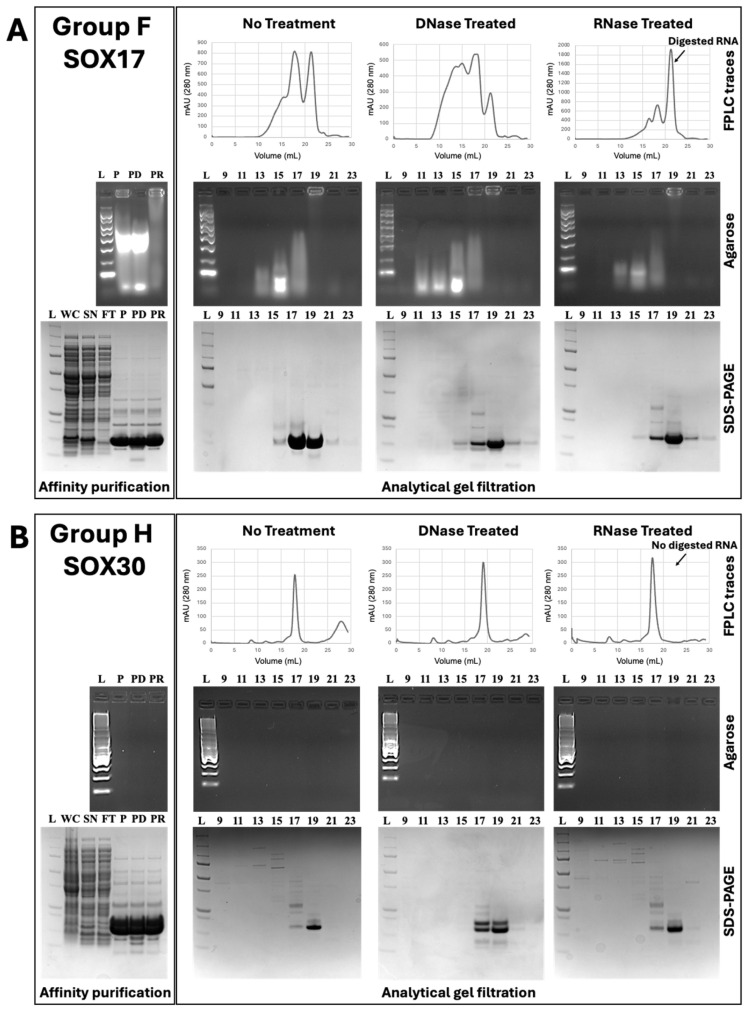
Comparison between purification profiles of SOX17 and SOX30 HMG-box domains. (**A**,**B**) SOX17 (Group F) and SOX30 (Group H) HMG-box domains were first purified via affinity chromatography (left panel). Following affinity purification, proteins were either left untreated or treated with DNase or RNase, before further purification via analytical gel filtration (right panel). Gel samples were taken of whole cell (WC), supernatant (SN), flowthrough (FT), purified eluant (P), purified eluant treated with DNase (PD), and purified eluant treated with RNase (PR) and analysed on agarose gels, for visualisation of nucleic acids, and via SDS-PAGE, for visualisation of protein. L = ladder. (**A**) The SOX17 HMG-box domain co-purifies with RNA during affinity and analytical gel filtration chromatography. In no treatment and DNase-treated samples, SOX17 HMG-box elutes around 17 to 19 mL, and RNA can be detected in fractions 10 to 19. In RNase-treated samples, the RNA-related peak shifts to fraction 22. As visualised on the agarose gels, the majority of the nucleic acids were attributed to RNA, with the least seen following RNase treatment. (**B**) The SOX30 HMG-box domain does not co-purify with RNA. In no treatment, DNase-treated, and RNase-treated samples, SOX30 HMG-box elutes around 17 to 19 mL, with no RNA detected in any of the fractions. Further, no nucleic acids are detected on the agarose gels.

We found that the SOX17 HMG-box domain (group F) co-purified with a large amount of RNA (Figure 2A). While some of the SOX17:RNA complex dissociated during analytical gel filtration, a proportion of SOX17 co-eluted with RNA. Treatment with DNase and RNase confirmed that the majority of the bound nucleic acid was RNA, since treatment with RNase removed most of the absorbance associated with fractions 9–16 and shifted the RNA peak towards the end of the elution profile, indicative of digested RNA, and resulted in the least nucleic acids visible on the agarose gels. The majority of the absorbance on the analytical gel filtration profiles could be attributed to RNA, based on the large second peak that appeared upon RNase treatment which was almost three times greater than the peak associated with SOX17. Experiments performed with SRY (group A), SOX2 (group B1), SOX21 (group B2), SOX11 (group C), SOX6 (group D), SOX9 (group E), and SOX15 (group G) HMG-box domains all similarly co-purified with RNA (Figures S1–S7). Interestingly, we found that the SOX30 HMG-box domain (group H) did not co-purify with any RNA, with all analytical gel filtration profiles appearing very similar for no treatment, RNase-treated, and DNase-treated samples, as well as the absence of any detectable nucleic acid in the agarose gels (Figure 2B).

In summary, representative SOX proteins from each group, with the exception of group H (SOX30), bound RNA. While some SOX proteins bound large amounts of RNA, to the extent that the RNA peak on the analytical gel filtration profile surpassed that of the protein following RNase treatment (SRY [group A], SOX2 [group B1], SOX21 [group B2], SOX11 [group C], SOX17 [group F], and SOX15 [group G]), others bound smaller amounts of RNA, with a greater protein peak than RNA peak (SOX6 [group D] and SOX9 [group E]). Finally, SOX30 (group H) showed no affinity for RNA.

### 3.2. The SOX HMG-Box Domain Interacts with ssDNA

**Figure 3:**
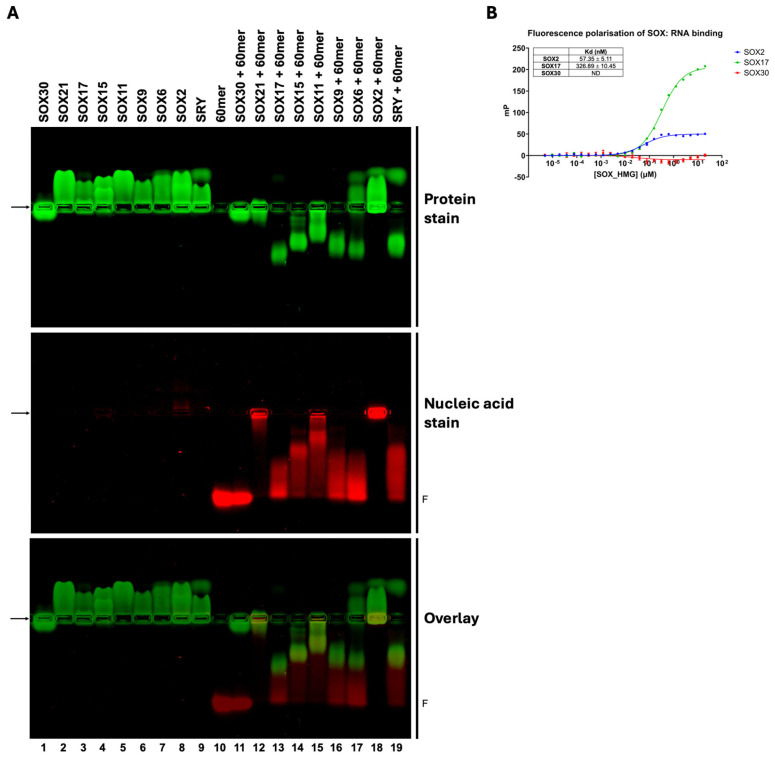
(**A**) EMSA results show that all SOX HMG-box domains tested, except for SOX30, alter the migration of a 60-mer ssDNA nucleic acid probe, indicating direct binding. Proteins were stained with Coomassie blue (top panel; green), the nucleic acid probe was stained with GelRed (middle panel; red), and the overlay is displayed in the bottom panel, with the complexes shown in dark green. Arrows indicate sample loading position; F indicates free 60-mer nucleic acid probe. (**B**) Fluorescence polarisation assays measuring binding affinity between SOX proteins and a FAM-labelled RNA probe verified the EMSA results. SOX17 (green) bound RNA with a Kd of ~327 nM, with no RNA binding detected for SOX30 (red). SOX2 (blue) was run as a positive control and bound RNA with a Kd of ~57 nM. Data shown as *n* = 3; error bars represent mean ± standard error of the mean; ND = not determined.

To further examine the RNA binding properties of SOX family members and establish whether there is a direct binding interaction, each of the SOX proteins were purified free of nucleic acids by nuclease treatment and subsequent purification steps. To confirm that all purified protein was free of nucleic acids, we ran the protein alone on an agarose gel, as well as spectrophotometrically confirmed the presence of pure protein using an absorbance ratio of 260/280, with a value of 0.7 indicating pure protein and a protein/RNA mixture typically with values of 1.7. We then tested whether these proteins were able to bind a 60-mer ssDNA nucleic acid probe via EMSA (Figure 3A). We found that both SRY and the probe shifted and co-migrated, indicating direct binding of the SRY:60-mer complex. Both SOX2 and SOX21 also shifted the probe, indicating direct binding; however, these SOX:60-mer complexes failed to migrate from the well, potentially due to decreased solubility upon complex formation. SOX6, SOX9, SOX11, SOX15, and SOX17 all exhibited altered migration paths of the protein and the probe, similarly indicating direct binding. In agreement with our observation that SOX30 failed to bind RNA, SOX30 was unable to alter migration of the probe, indicating no direct binding. To validate what was shown with ssDNA, fluorescence polarisation was utilised to measure binding affinity between SOX proteins and a FAM-labelled RNA probe previously shown to bind SOX2 [39]. SOX2 bound RNA with high affinity (Kd ~57 nM), consistent with previous reports [39]. Compared to the SOX2 control, SOX17 (representative RNA binding SOX protein) bound RNA with ~6-fold weaker affinity at a Kd of ~327 nM, while no RNA binding was detected for SOX30 (Figure 3B).

### 3.3. The C-Terminal Region of the SOX17 HMG-Box Domain Is Responsible for RNA Interaction

**Figure 4:**
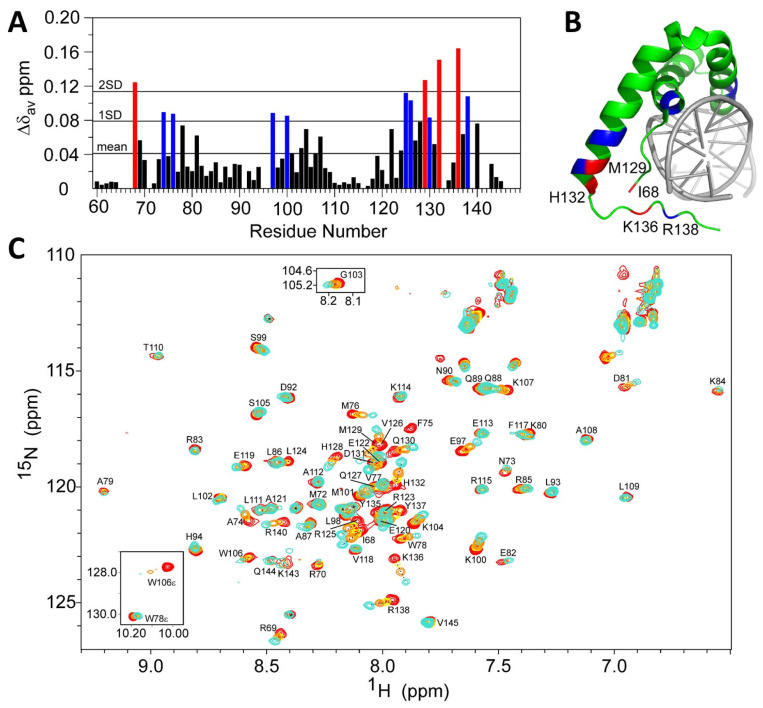
^15^N,^1^H HSQC-monitored NMR titration of ^15^N-labelled SOX17 HMG-box domain indicates the importance of the C-terminal residues for binding a 12-mer ssDNA nucleic acid probe. (**A**) Plot of the change in average ^1^HN and ^15^N chemical shifts (blue indicates residues with 1 standard deviation (SD) of the mean of chemical shift; red indicates residues with 2 SD). More shift in a residue means more conformational change in the interaction with the nucleic acid probe. (**B**) The crystal structure of the SOX17 HMG-box domain bound to DNA (PDB ID: 3F27) [58], highlighting the position of residues with significant chemical shifts (blue indicates residues with 1 SD of the mean of chemical shift; red indicates residues with 2 SD). (**C**) ^1^H,^15^N HSQC spectrum indicating chemical shift dependence on the presence of a 12-mer ssDNA nucleic acid probe. Red (no ssDNA), yellow (25 µM ssDNA), orange (50 µM ssDNA), and cyan (100 µM ssDNA).

To identify the regions responsible for RNA interaction, we performed crystallographic and NMR experiments with the SOX17 HMG-box domain, which was selected due to its obvious RNA/ssDNA binding ability, as demonstrated in the analytical gel filtration purification, EMSA, and fluorescence polarisation data. Whilst the crystallographic approach failed to produce diffracting crystals, NMR was able to identify key shifts in the ^15^N,^1^H HSQC spectra of ^15^N-labelled SOX17 upon titration (1:1) with a 12-mer ssDNA nucleic acid probe (Figure 4). Significant chemical shift changes were observed for the C-terminal region (Arg125 to Arg138), the N-terminal region (Ile68, Ala74, and Met76), and residues in the central helix (Glu97 and Lys100). The indole signal of Trp106 also shifted and significantly broadened on titration with the probe.

**Figure 5:**
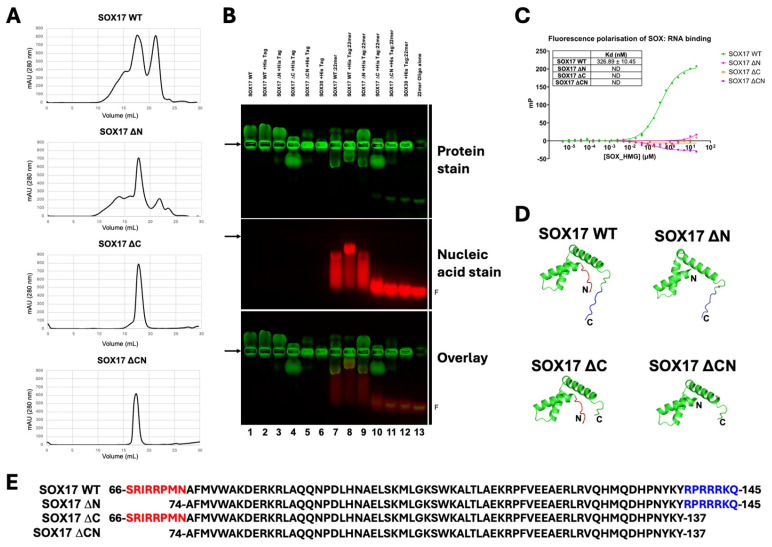
The C-terminal region of the SOX17 HMG-box domain is critical for RNA binding. (**A**) Analytical gel filtration profiles of SOX17 wild-type (SOX17 WT), N-terminal truncation (SOX17 ∆N), C-terminal truncation (SOX17 ∆C), and N- and C-terminal truncation (SOX17 ∆CN) HMG-box domain constructs. Co-purification of RNA is observed with SOX17 WT and SOX17 ∆N, while no RNA co-purification is evident with SOX17 ∆C or SOX17 ∆CN. (**B**) EMSA between WT and truncated SOX17 constructs and a 22-mer ssDNA nucleic acid probe; SOX30 was used as a negative control. SOX17 WT and SOX17 ∆N can bind to the nucleic acid probe and shift its position, while C-terminal truncation of the HMG-box domain abolishes RNA binding, as evident in SOX17 ∆C and SOX17 ∆CN. Arrows indicate sample loading position; F indicates free 22-mer nucleic acid probe. (**C**) Fluorescence polarisation assays measuring binding affinity between WT and truncated SOX17 proteins and a FAM-labelled RNA probe. SOX17 WT (green) bound RNA with a Kd of ~327 nM, with no RNA binding detected for SOX17 ∆C (orange) or SOX17 ∆CN (purple). Some RNA binding was detected at the highest concentrations of SOX17 ∆N (pink); however, the binding affinity was too low to be determined. SOX17 WT data as shown in Figure 3B. Data shown as *n* = 3; error bars represent mean ± standard error of the mean; ND = not determined. (**D**) Models of the SOX17 HMG-box domain in SOX17 WT, SOX17 ∆N, SOX17 ∆C, and SOX17 ∆CN constructs. The N-terminal truncated region is shown in red; the C-terminal truncated region is shown in blue. (**E**) Sequences of the WT and truncated SOX17 HMG-box domain constructs used, numbered as per full-length UniProt sequences.

**Figure 6:**
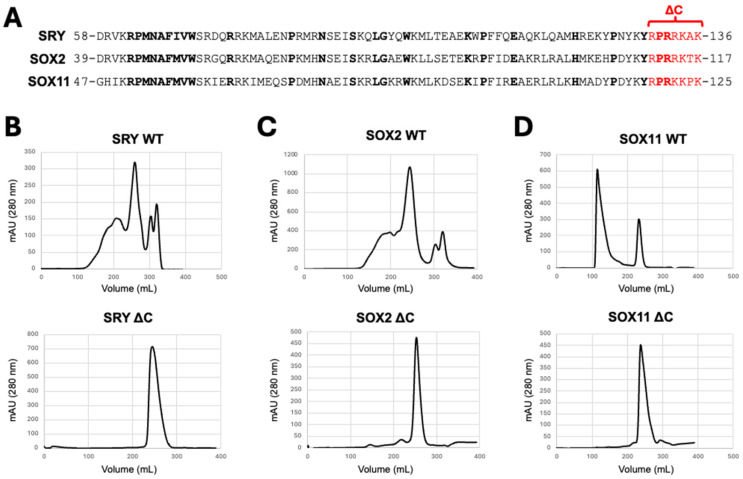
The C-terminal region of the HMG-box domains of SRY, SOX2, and SOX11 similarly play an important role in RNA binding. (**A**) Aligned amino acid sequences of SRY, SOX2, and SOX11 HMG-box domains, numbered as per full-length UniProt sequences. Conserved residues are shown in bold. Residues removed in C-terminal truncation (∆C) constructs, proposed to be critical for RNA binding, are shown in red. (**B**–**D**) SEC graphs of HMG-box domain wild-type (WT) and ∆C constructs of SRY (**B**), SOX2 (**C**), and SOX11 (**D**). SRY WT, SOX2 WT, and SOX11 WT all co-purify with RNA, while SRY ∆C, SOX2 ∆C, and SOX11 ∆C do not, demonstrating that C-terminal truncation of the HMG-box domain disrupts RNA binding.

Due to the large number of chemical shift changes in the N- and C-terminal regions, we designed a series of mutants with N- and/or C-terminal truncations (denoted as ∆) of the SOX17 HMG-box domain (Figure 5; Table S3), only removing residues outside of the α-helices. We found that while wild-type (WT) SOX17 and SOX17 ∆N bound RNA via analytical gel filtration and were able to shift migration of a ssDNA nucleic acid probe, SOX17 ∆C and SOX17 ∆CN mutants were unable to (Figure 5A,B). We also measured binding affinity between SOX17 truncation proteins and a FAM-labelled RNA probe, with SOX17 ∆C and SOX17 ∆CN abolishing RNA binding compared to SOX17 WT. Some RNA binding was detected at the highest concentrations of SOX17 ∆N; however, the binding affinity was too low to be determined (Figure 5C). This further indicates the importance of the C-terminal region, specifically the seven amino acid region 138-RPRRRKQ-144 (73–79 HMG-box numbering), in the RNA binding ability of the SOX HMG-box domain. To investigate whether these deletions could similarly affect other SOX family members, we also assessed whether SRY ∆C, SOX2 ∆C, and SOX11 ∆C mutants were able to bind RNA, finding that in all cases, removal of the seven amino acid C-terminal region (73–79 HMG-box numbering) prevented RNA binding (Figure 6).

### 3.4. SOX30 Retains a Structured HMG-Box Domain

Due to its inability to bind RNA, we sought to characterise the structure of the SOX30 HMG-box domain. The protein was recombinantly expressed, purified, and crystallised. Crystals formed in 0.1 M sodium acetate, 2 M ammonium sulphate, pH 4.6, and diffracted to 1.4 Å resolution. The diffraction data were indexed and integrated in the space group *P*2_1_2_1_2_1_. The structure was solved by molecular replacement in Phaser using the α-helices of SOX2 as the reference model (PDB ID: 1O4X) [55], followed by rebuilding in COOT and refinement in Phenix [56] (see Table 1 for data collection and refinement statistics). The structure was deposited to the Protein Data Bank with PDB ID: 7JJK.

**Figure 7:**
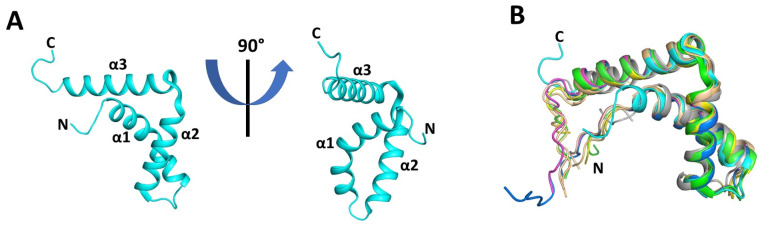
Crystal structure of the SOX30 HMG-box domain and comparison with other SOX family member HMG-box domain structures. (**A**) Structure of the SOX30 HMG-box domain (residues 335–405) at 0° and 90°, showing the typical three α-helices arranged in an L-shape and flanked by two disordered regions at the N- and C-termini. (**B**) Superimposition of the SOX30 HMG-box domain with other SOX protein HMG-box domains, demonstrating a very similar secondary structure between SOX family members. The seven amino acids identified as critical for RNA binding (73–79, HMG-box numbering) are located in the disordered C-terminal end of the HMG-box domain and are thus not visible in the SOX30 structure or the structures of Sox5, SOX9, and SOX17. SOX30 is shown in cyan; Sox4 is shown in dark orange (PDB ID: 3U2B); Sox18 is shown in light green (PDB ID: 4Y60); SOX11 is shown in light orange (PDB ID: 6T78); SOX2 is shown in magenta (PDB ID: 1O4X); SRY is shown in blue (PDB ID: 1J46); SOX17 is shown in green (PDB ID: 4A3N); SOX9 is shown in yellow (PDB ID: 4EUW); Sox5 is shown in grey (PDB ID: 1I11).

The crystal structure revealed that the SOX30 HMG-box domain contains the typical features of an HMG-box, including three α-helices and two disordered regions towards the N- and C-termini. The three α-helices form an L-shape in which α1 and α2 create the major wing and α3 makes the minor wing (Figure 7A). Superimposing our SOX30 HMG-box domain structure on other available SOX protein HMG-box domain structures (alone and DNA-bound) demonstrated a very similar secondary structure between SOX family members (Figure 7B), with a low RMSD (Table 2). The largest differences between the superimposed SOX structures are observed at the N- and C-termini, which is to be expected given that these regions are disordered and adopt multiple conformations. The C-terminal end of the SOX30 structure is seen to be orientated in a different direction to the other SOX structures. However, this is due to differences in crystal packing, with the SOX30 molecule within the asymmetric unit sandwiched between two adjacent SOX30 molecules, forming crystal contacts that stabilise the C-terminal end in this conformation. The seven amino acids at the C-terminal end of the HMG-box that we identified as critical for RNA binding (407-QPRPGKR-413; 73–79 HMG-box numbering) are not visible in the SOX30 structure. This critical binding region is located within the disordered C-terminal end of the HMG-box domain, and thus is often not visible in structures, including those of Sox5, SOX9, and SOX17. Regardless, the structural similarities in the remainder of the SOX30 HMG-box support our claim that this C-terminal end is key for RNA binding.

Structurally, the HMG-box domain of SOX30 retained all of the key features of other SOX proteins, with no obvious structural differences that would indicate why SOX30 does not bind RNA. Inspection of the C-terminal region of the SOX30 HMG-box domain, shown here to be responsible for RNA binding in other SOX family members, revealed sequence differences that are distinct from other SOX proteins. As shown in Figure 1, the final five C-terminal residues in the HMG-box domain (residues 75–79, HMG-box numbering) feature a consensus sequence of Rrkkk, thus containing a strong clustering of positive residues. Conversely, SOX30 has the sequence RPGKR, and thus has lost 40% of its positive charge within this cluster, which may contribute to the loss of RNA binding ability.

In the present study, we demonstrate that the HMG-box domains of representatives of all SOX groups, with the exception of SOX30 (group H), bind RNA. While the DNA binding capability and function of SOX proteins have been well characterised, including detailed structural approaches [59,60,61,62,63,65], little is known as to how SOX proteins interact with RNA. Here, we show that the disordered C-terminal region of the HMG-box domain of SOX proteins is critical for RNA binding. Our NMR studies indicate that although chemical shifts can be observed across a range of residues within the HMG-box of SOX17, the shift is significantly greater in the basic-rich C-terminal region. Consistent with SOX17 data, we also show that C-terminal truncation of the HMG-box domains of SRY, SOX2, and SOX11 result in a dramatic reduction in RNA binding.

The consensus sequence of the C-terminal tail of the HMG-box domain of SOX proteins (70-ykYrPRrkkk-79, Figure 1) may provide insights into the differences in RNA binding ability of SOX proteins. SOX family members that bound larger amounts of RNA (SRY, SOX2, SOX21, SOX11, SOX17, and SOX15) feature the common sequence 70-YKYRPRR/K-76, whereas SOX6 and SOX9 that bound smaller amounts of RNA have the sequences 70-YKYKPRP-76 and 70-YKYQPRR-76, respectively. Conversely, SOX30, which shows no RNA binding, features the sequence 70-WVYQPRP-76. As the PR motif (74–75) is conserved throughout all SOX proteins, it is not the sole RNA-binding determinant. The R/K residues flanking this motif (73/76) enhance, but are not strictly required for, RNA binding, as group D and E SOX proteins lack these residues yet still (weakly) bind RNA. Residues 70-YKY-72 upstream of the PR motif are conserved in all SOX proteins except for SOX30 and may influence RNA binding; however, they are also not the sole binding determinant as △C truncations retaining these residues still lost RNA binding ability. In addition, SOX30 is the only protein lacking a basic residue at position 77 (K/R > G). As such, it is likely that multiple residues within the HMG-box domain C-terminus are required to confer RNA binding.

A 60-amino acid region directly after the HMG-box has previously been linked to the RNA binding ability of Sox2 [42,43]. This differs from our data that identify a seven amino acid region at the C-terminal end of the SOX protein HMG-box (73-rPRrkkk-79; Figure 1, black dashed box) as being critical for RNA binding; however, our study was restricted to the HMG-box only and did not investigate regions outside of this. Another study linked the RNA binding ability of SOX2 to an ARM-like domain encompassing the C-terminal end of the HMG-box and residues directly after this, with EMSA analysis of RNA with a peptide encoding R/K > A mutations of the SOX2-ARM demonstrating abolished RNA binding [43]. The region identified in this study includes the seven amino acids we found to be critical for RNA binding, confirming the importance of basic residues in this region for RNA binding of SOX proteins. This region found to be necessary for RNA binding has also been shown to be involved in DNA binding, as well as interactions with importins that drive nuclear import [14]. Whilst speculative, this competition between important cellular binding partners may play a role in the ability of SOX proteins to differentially regulate development over a wide range of cell and tissue types. While further detailed experiments will be required to elucidate this, our study provides an important insight into the regions that can be targeted to dissect such interactions and the important biological functions of SOX proteins.

## Figures and Tables

**Table 1:** Data collection and refinement statistics. Statistics for the highest resolution shell are shown in parentheses.

Data Collection and Processing	SOX30 HMG-Box
Wavelength (Å)	0.95372
Resolution range (Å)	17.99–1.4 (1.4–1.42)
Space group	*P*2_1_2_1_2_1_
Unit cell (Å, °)	33.7, 35.7, 52.91, 90, 90, 90
Unique reflections	13155 (656)
Multiplicity	11.8 (9.9)
Completeness (%)	99.9 (99.0)
Mean I/sigma(I)	17.8 (4.4)
Wilson B-factor Å^2^	11.04
Rpim	0.029 (0.19)
CC(1/2)	0.999 (0.968)
Refinement	
Number of reflections	13111
Number of R-free reflections	626
R-work (%)	17.37
R-free (%)	19.92
RMS(bonds)	0.0093
RMS(angles)	2.583
Ramachandran plot	
favoured (%)	100
allowed (%)	0
outliers (%)	0
PDB accession code	7JJK

**Table 2:** SOX protein HMG-box domain structures and their similarity to the SOX30 HMG-box domain structure. RMSD calculated for the region encompassing helices α1 to α3.

Protein	PDB ID (Reference)	Resolution (Å)	RMSD to SOX30 (Å)
Sox4	3U2B [59]	2.40	0.613 [over 47 Cα]
SOX11	6T78 [60]	2.50	0.676 [over 47 Cα]
SOX2	1O4X [61]	NMR	0.726 [over 49 Cα]
SRY	1J46 [62]	NMR	0.755 [over 52 Cα]
Sox18	4Y60 [63]	1.75	0.766 [over 54 Cα]
SOX9	4EUW [64]	2.77	0.918 [over 52 Cα]
SOX17	4A3N [65]	2.40	0.925 [over 53 Cα]
Sox5	1I11 [66]	NMR	1.350 [over 49 Cα]

## Data Availability

Files associated with the structure generated in this study have been deposited to the Protein Data Bank and were released prior to submission of the manuscript with PDB ID: 7JJK. Source data are provided with the paper.

## Supplementary Materials

The following supporting information can be downloaded at: https://www.mdpi.com/article/10.3390/cells13141202/s1, Figure S1: The SRY HMG-box domain co-purifies with RNA during affinity and size exclusion chromatography; Figure S2: The SOX2 HMG-box domain co-purifies with RNA during affinity and size exclusion chromatography; Figure S3: The SOX21 HMG-box domain co-purifies with RNA during affinity and size exclusion chromatography; Figure S4: The SOX11 HMG-box domain co-purifies with RNA during affinity and size exclusion chromatography; Figure S5: The SOX6 HMG-box domain co-purifies with RNA during affinity and size exclusion chromatography; Figure S6: The SOX9 HMG-box domain co-purifies with RNA during affinity and size exclusion chromatography; Figure S7: The SOX15 HMG-box domain co-purifies with RNA during affinity and size exclusion chromatography; Table S1: SOX HMG-box domain constructs; Table S2: Nucleic acid binding substrate sequences; Table S3: SOX HMG-box domain mutant constructs.
