# Supplementary material for: RNA Binding Properties of SOX Family Members
Source: Cells. 2024 Jul 16;13(14):1202. doi: 10.3390/cells13141202 (PMC11274882; doi:10.3390/cells13141202)
Supplement: Supplementary file 1 [file cells-13-01202-s001.zip › cells-2549663-supplementary.pdf]

Supplementary Table S1: SOX HMG-box domain constructs

Protein	Uniprot ID	Residues	Plasmid	Resistance	Affinity tag	Description
SRY	Q05066	58-140	pMCSG21	Spectinomycin	X6 His	HMG-box domain
SOX2	P48431	39-117	pMCSG21	Spectinomycin	X6 His	HMG-box domain
SOX6	P35712	613-700	pMCSG21	Spectinomycin	X6 His	HMG-box domain
SOX9	P48436	103-183	pMCSG21	Spectinomycin	X6 His	HMG-box domain
SOX11	P35716	47-135	pET-30a(+)	Kanamycin	X6 His	HMG-box domain
SOX15	O60248	47-135	pET-30a(+)	Kanamycin	X6 His	HMG-box domain
SOX17	Q9H6I2	66-145	pMCSG21	Spectinomycin	X6 His	HMG-box domain
SOX21	Q9Y651	6-94	pET-30a(+)	Kanamycin	X6 His	HMG-box domain
SOX30	O94993	335-423	pET-30a(+)	Kanamycin	X6 His	HMG-box domain

Supplementary Table S2: Nucleic acid binding substrate sequences

Name	Sequence	Source/description
60-mer ssDNA	5'-GTTAGAGGTGCCCCACAGGCGGCGGTTAGTATTCCCCGCCGCCTGGGGCACCGGGGCAC-3'	Beak and feather disease virus loop construct
22-mer ssDNA	5'-CCCCATCTTAGTATATTAGTTA-3'	Random primer
12-mer ssDNA	5'-ATCGATCGATCG-3'	Short repeating sequence containing all nucleotides
40-mer RNA	5'-GGUCGCCGUGGCCACUUCGAAAGGGGUGGAAAGGGCGACC-FAM-3'	ES2 lncRNA hairpin "Loop A" [39]

Supplementary Table S3: SOX HMG-box domain mutant constructs

Protein	Uniprot ID	Residues	Plasmid	Resistance	Affinity tag	Description
SOX17 WT	Q9H6I2	66-145	pMCSG21	Spectinomycin	X6 His	HMG-box domain
SOX17 ΔN	Q9H6I2	74-145	pET-30a(+)	Kanamycin	X6 His	ΔN HMG-box domain
SOX17 ΔC	Q9H6I2	66-137	pET-30a(+)	Kanamycin	X6 His	ΔC HMG-box domain
SOX17 ΔCN	Q9H6I2	74-137	pET-30a(+)	Kanamycin	X6 His	ΔCN HMG-box domain
SRY ΔC	Q05066	58-129	pET-30a(+)	Kanamycin	X6 His	ΔC HMG-box domain
SOX2 ΔC	P48431	39-110	pET-30a(+)	Kanamycin	X6 His	ΔC HMG-box domain
SOX11 ΔC	P35716	47-118	pET-30a(+)	Kanamycin	X6 His	ΔC HMG-box domain

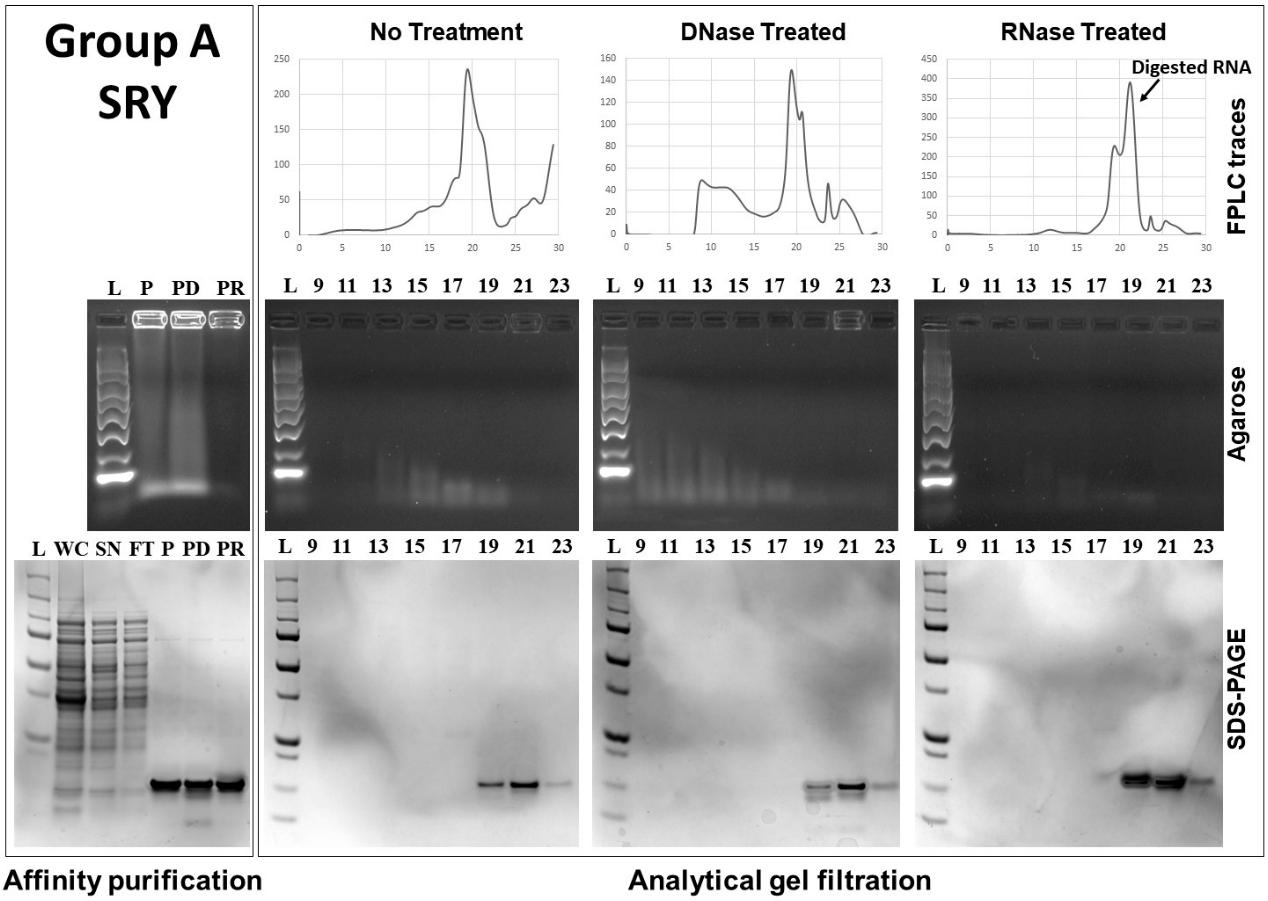

Supplementary Figure S1: The SRY HMG-box domain co-purifies with RNA during affinity and size exclusion chromatography. Left panel shows different stages during affinity chromatography. The FPLC trace, agarose gel, and SDS-PAGE analysis of the samples are indicated on the right. During purification, samples were taken for whole cell (WC), supernatant (SN), flowthrough (FT), purified eluant (P), purified eluant treated with DNase (PD), and purified eluant treated with RNase (PR). The right panel shows the subsequent analytical gel filtration of no treatment, DNase treated, and RNase treated SRY. In no treatment and DNase treated samples, SRY HMG-box elutes around 19 to 21 mL, and RNA can be detected in fractions 13 to 19. In RNase treated samples, the RNA-related peak shifts dramatically to fraction 22.

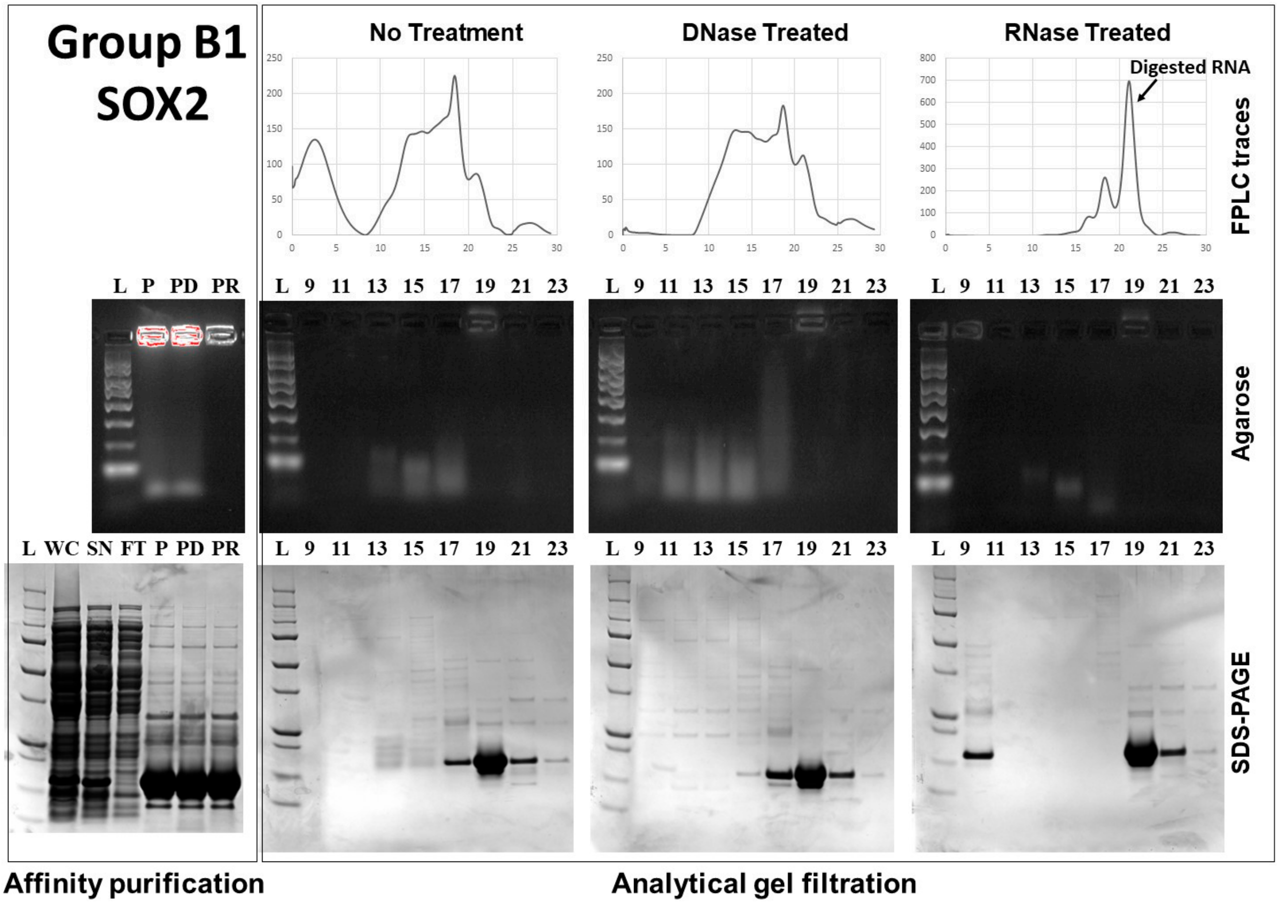

Supplementary Figure S2: The SOX2 HMG-box domain co-purifies with RNA during affinity and size exclusion chromatography. Left panel shows different stages during affinity chromatography. The FPLC trace, agarose gel, and SDS-PAGE analysis of the samples are indicated on the right. During purification, samples were taken for whole cell (WC), supernatant (SN), flowthrough (FT), purified eluant (P), purified eluant treated with DNase (PD), and purified eluant treated with RNase (PR). The right panel shows the subsequent analytical gel filtration of no treatment, DNase treated, and RNase treated SOX2. In no treatment and DNase treated samples, SOX2 HMG-box elutes around 19 to 21 mL, and RNA can be detected in fractions 10 to 19. In RNase treated samples, the RNA-related peak shifts to fraction 22.

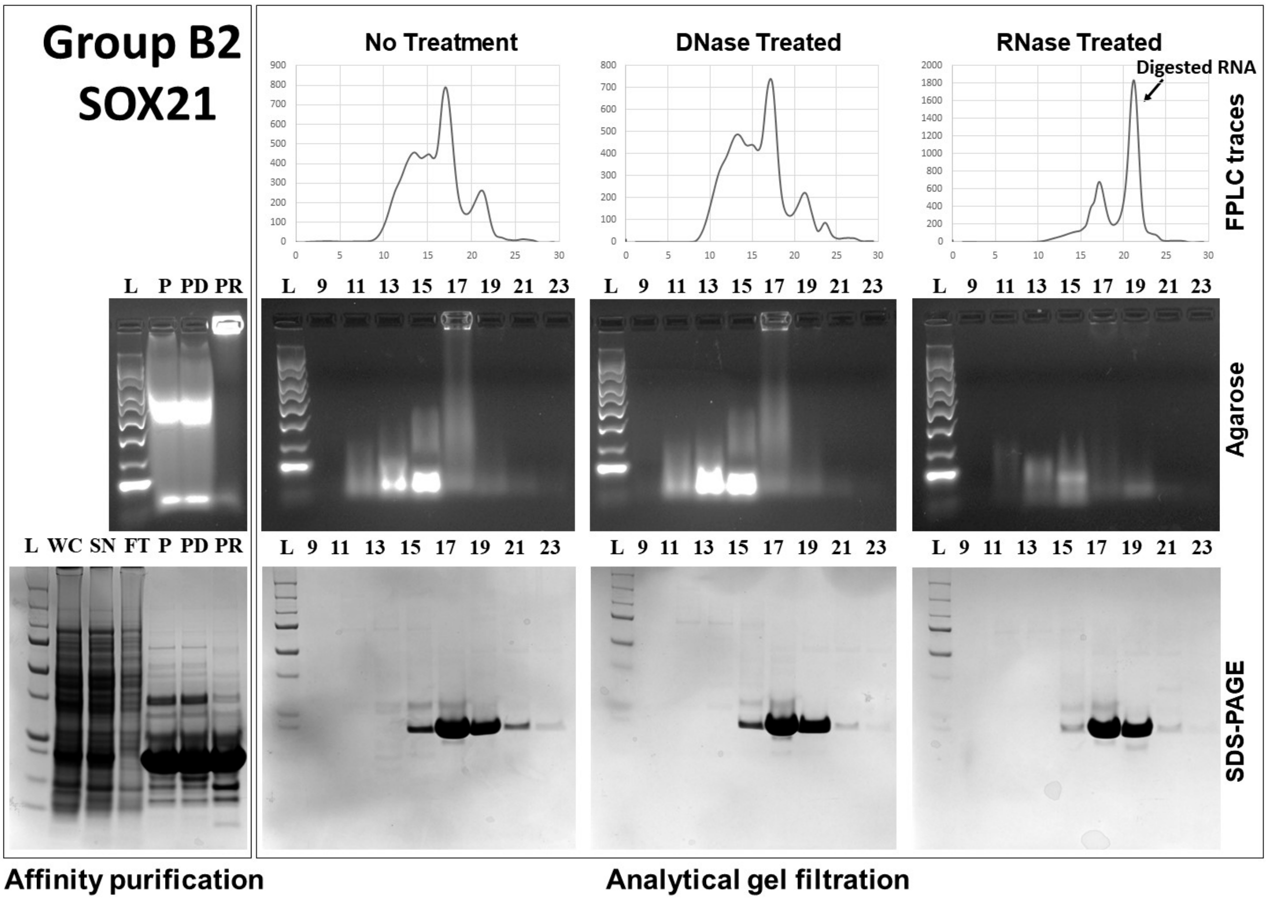

Supplementary Figure S3: The SOX21 HMG-box domain co-purifies with RNA during affinity and size exclusion chromatography. Left panel shows different stages during affinity chromatography. The FPLC trace, agarose gel, and SDS-PAGE analysis of the samples are indicated on the right. During purification, samples were taken for whole cell (WC), supernatant (SN), flowthrough (FT), purified eluant (P), purified eluant treated with DNase (PD), and purified eluant treated with RNase (PR). The right panel shows the subsequent analytical gel filtration of no treatment, DNase treated, and RNase treated SOX21. In no treatment and DNase treated samples, SOX21 HMG-box elutes around 17 to 19 mL, and RNA can be detected in fractions 10 to 17. In RNase treated samples, the RNA-related peak shifts to fraction 22.

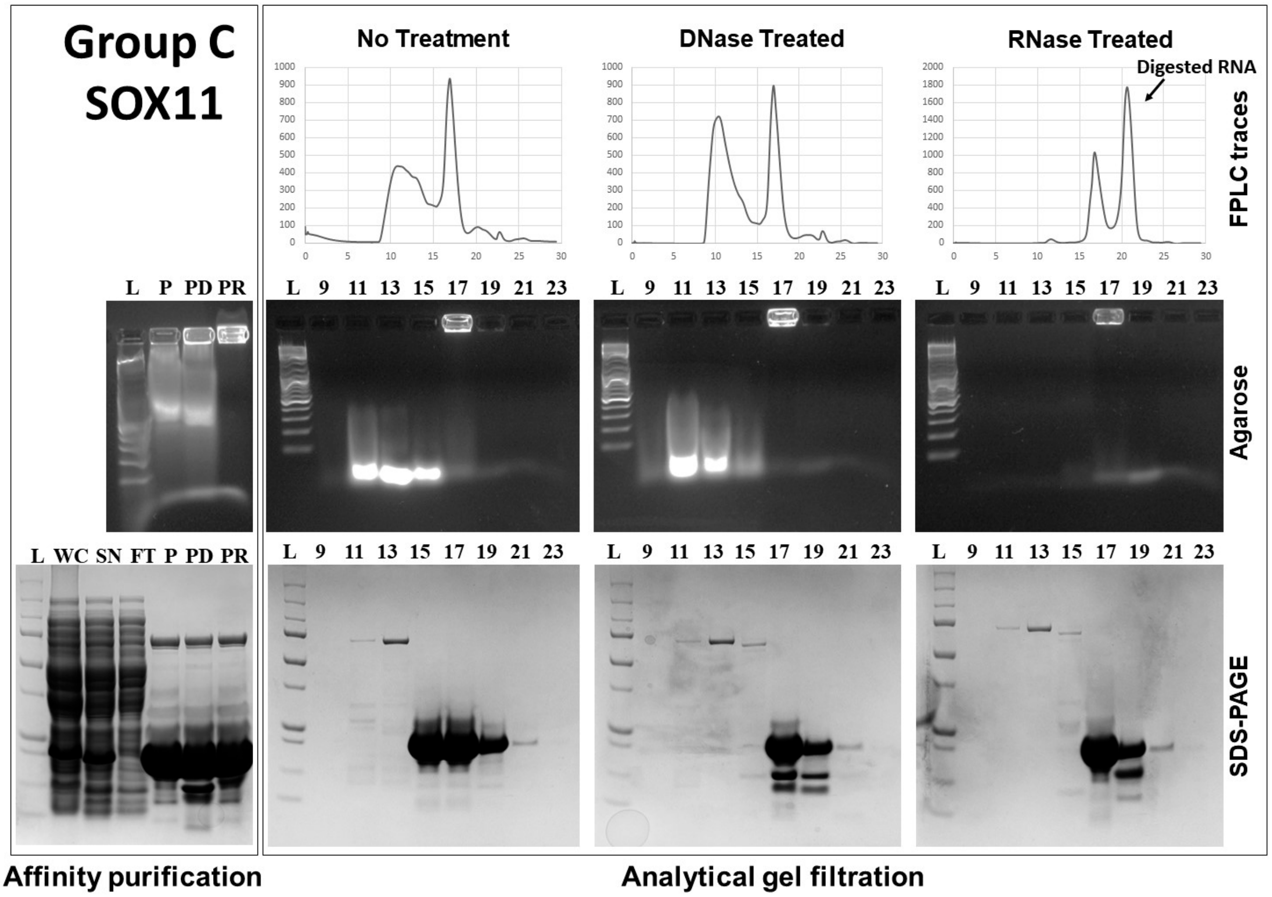

Supplementary Figure S4: The SOX11 HMG-box domain co-purifies with RNA during affinity and size exclusion chromatography. Left panel shows different stages during affinity chromatography. The FPLC trace, agarose gel, and SDS-PAGE analysis of the samples are indicated on the right. During purification, samples were taken for whole cell (WC), supernatant (SN), flowthrough (FT), purified eluant (P), purified eluant treated with DNase (PD), and purified eluant treated with RNase (PR). The right panel shows the subsequent analytical gel filtration of no treatment, DNase treated, and RNase treated SOX11. In no treatment and DNase treated samples, SOX11 HMG-box elutes around 17 to 19 mL, and RNA can be detected in fractions 9 to 16. In RNase treated samples, the RNA-related peak shifts to fraction 21.

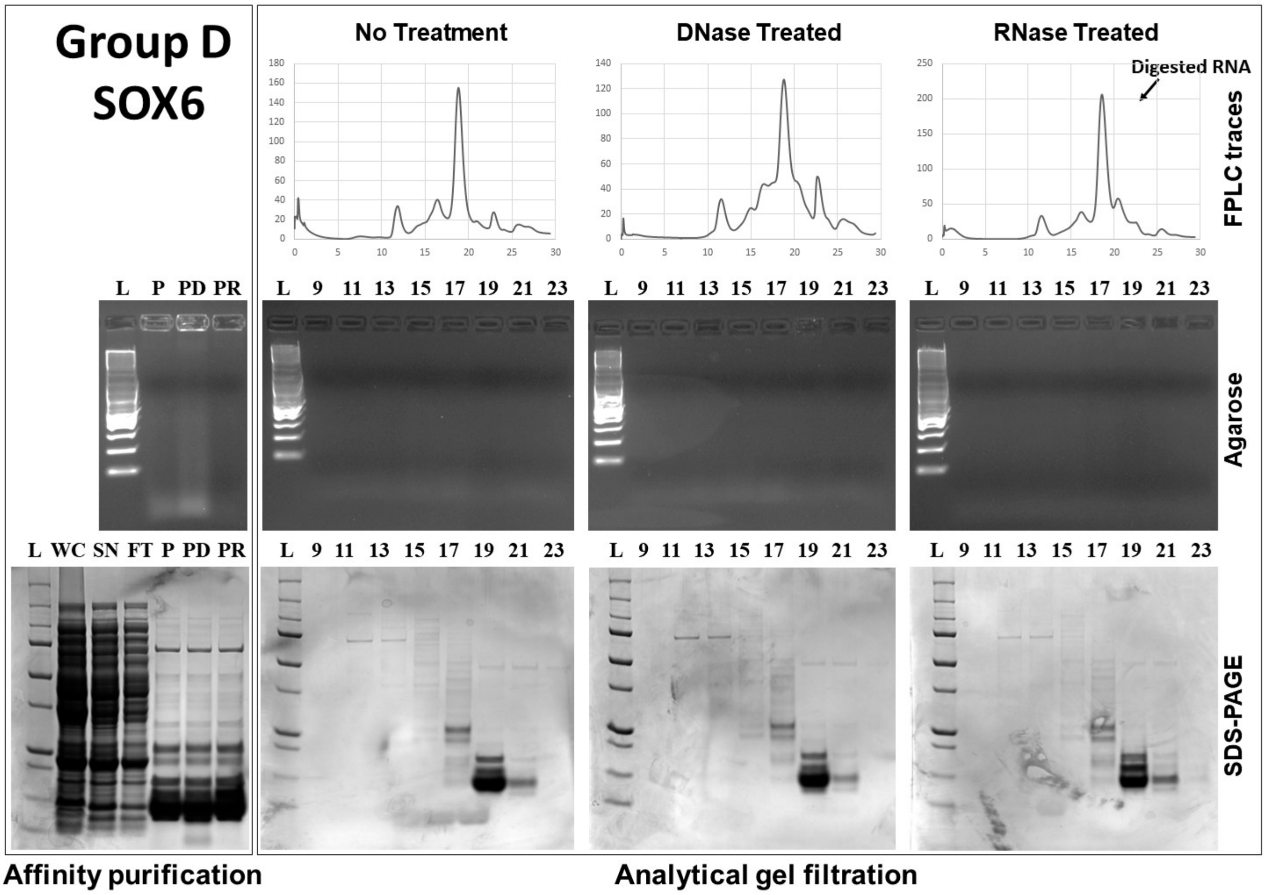

Supplementary Figure S5: The SOX6 HMG-box domain co-purifies with RNA during affinity and size exclusion chromatography. Left panel shows different stages during affinity chromatography. The FPLC trace, agarose gel, and SDS-PAGE analysis of the samples are indicated on the right. During purification, samples were taken for whole cell (WC), supernatant (SN), flowthrough (FT), purified eluant (P), purified eluant treated with DNase (PD), and purified eluant treated with RNase (PR). The right panel shows the subsequent analytical gel filtration of no treatment, DNase treated, and RNase treated SOX6. While co-purified RNA can be seen in affinity purification samples, the RNA bands are very faint in the gel filtration samples.

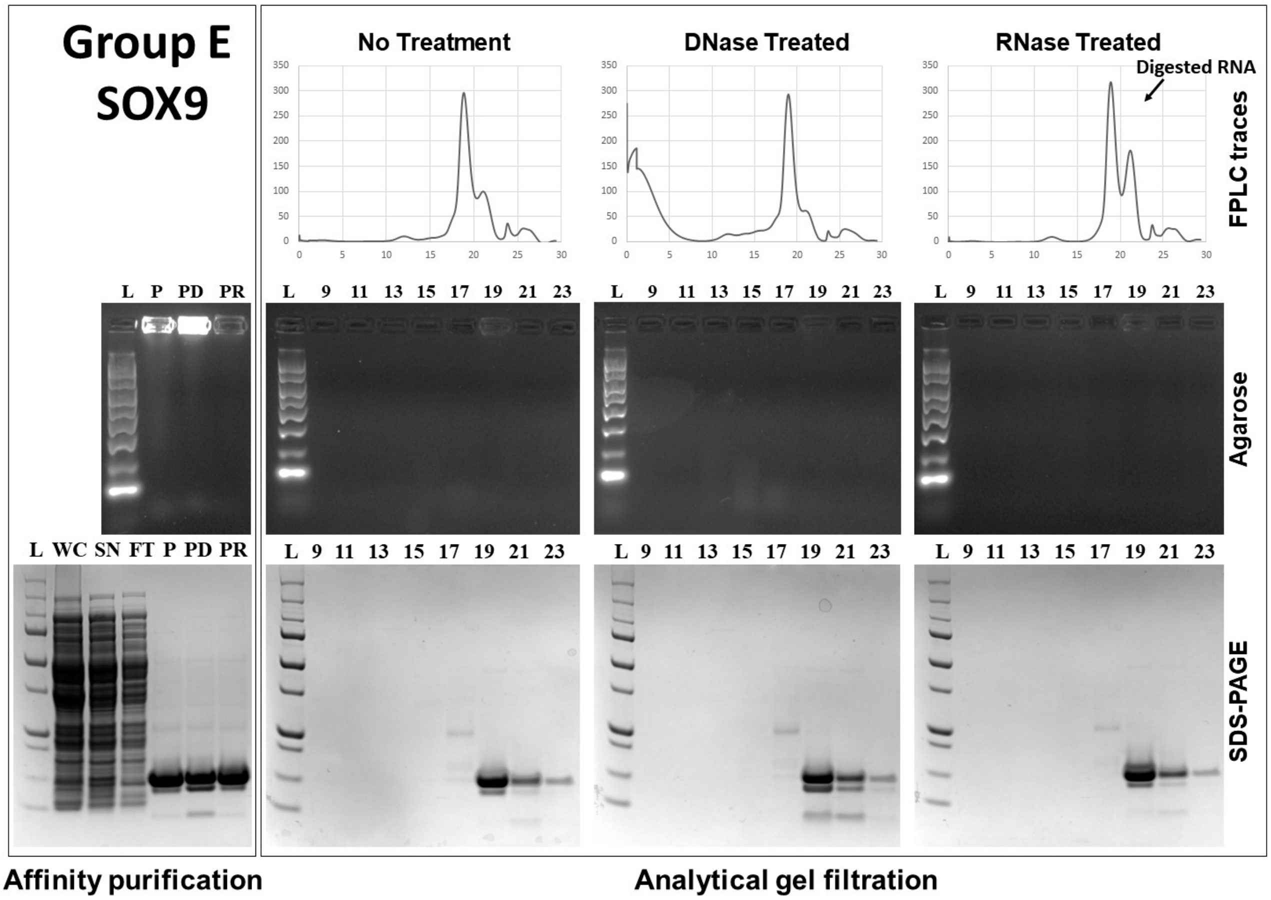

Supplementary Figure S6: The SOX9 HMG-box domain co-purifies with RNA during affinity and size exclusion chromatography. Left panel shows different stages during affinity chromatography. The FPLC trace, agarose gel, and SDS-PAGE analysis of the samples are indicated on the right. During purification, samples were taken for whole cell (WC), supernatant (SN), flowthrough (FT), purified eluant (P), purified eluant treated with DNase (PD), and purified eluant treated with RNase (PR). The right panel shows the subsequent analytical gel filtration of no treatment, DNase treated, and RNase treated SOX9. While co-purified RNA can be seen in affinity purification samples, the RNA bands are very faint in the gel filtration samples.

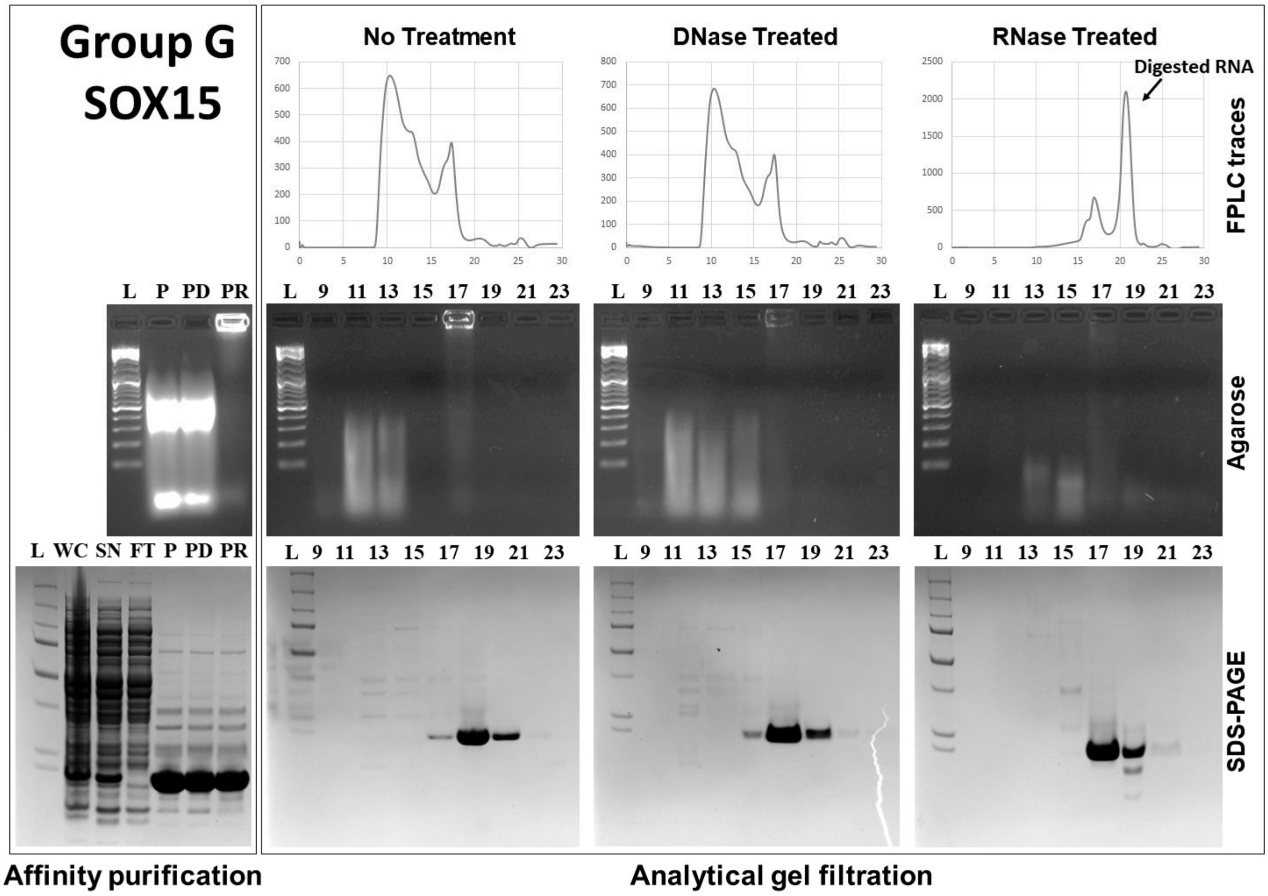

Supplementary Figure S7: The SOX15 HMG-box domain co-purifies with RNA during affinity and size exclusion chromatography. Left panel shows different stages during affinity chromatography. The FPLC trace, agarose gel, and SDS-PAGE analysis of the samples are indicated on the right. During purification, samples were taken for whole cell (WC), supernatant (SN), flowthrough (FT), purified eluant (P), purified eluant treated with DNase (PD), and purified eluant treated with RNase (PR). The right panel shows the subsequent analytical gel filtration of no treatment, DNase treated, and RNase treated SOX15. In no treatment and DNase treated samples, SOX15 HMG-box elutes around 17 to 19 mL, and RNA can be detected in fractions 9 to 16. In RNase treated samples, the RNA-related peak shifts to fraction 22.

References:

39. Holmes, Z.E.; Hamilton, D.J.; Hwang, T.; Parsonnet, N.V.; Rinn, J.L.; Wuttke, D.S.; Batey, R.T. The Sox2 transcription factor binds RNA. *Nat. Commun.* **2020**, *11*, 1-12.
